# Engineering the vaccinia virus L1 protein for increased neutralizing antibody response after DNA immunization

**DOI:** 10.1186/1743-422X-6-28

**Published:** 2009-03-03

**Authors:** Kaori Shinoda, Linda S Wyatt, Kari R Irvine, Bernard Moss

**Affiliations:** 1 Laboratory of Viral Diseases, National Institute of Allergy and Infectious Diseases, National Institutes of Health, Bethesda, Maryland 20892-3210, USA

## Abstract

**Background:**

The licensed smallpox vaccine, comprised of infectious vaccinia virus, has associated adverse effects, particularly for immunocompromised individuals. Therefore, safer DNA and protein vaccines are being investigated. The L1 protein, a component of the mature virion membrane that is conserved in all sequenced poxviruses, is required for vaccinia virus entry into host cells and is a target for neutralizing antibody. When expressed by vaccinia virus, the unglycosylated, myristoylated L1 protein attaches to the viral membrane via a C-terminal transmembrane anchor without traversing the secretory pathway. The purpose of the present study was to investigate modifications of the gene expressing the L1 protein that would increase immunogenicity in mice when delivered by a gene gun.

**Results:**

The L1 gene was codon modified for optimal expression in mammalian cells and potential N-glycosylation sites removed. Addition of a signal sequence to the N-terminus of L1 increased cell surface expression as shown by confocal microscopy and flow cytometry of transfected cells. Removal of the transmembrane domain led to secretion of L1 into the medium. Induction of binding and neutralizing antibodies in mice was enhanced by gene gun delivery of L1 containing the signal sequence with or without the transmembrane domain. Each L1 construct partially protected mice against weight loss caused by intranasal administration of vaccinia virus.

**Conclusion:**

Modifications of the vaccinia virus L1 gene including codon optimization and addition of a signal sequence with or without deletion of the transmembrane domain can enhance the neutralizing antibody response of a DNA vaccine.

## Background

Since the eradication of smallpox and the cessation of vaccination three decades ago, large segments of the population have become susceptible to infection with variola virus [1]. This vulnerability coupled with fears of variola virus dissemination for nefarious purposes have led to a resurgence of interest in smallpox vaccination [2,3]. The current smallpox vaccine consists of infectious vaccinia virus (VACV), which is closely related to variola virus, and provides complete and long lasting immunity [4]. Nevertheless, the live vaccine can produce serious side effects particularly in individuals with an immunodeficiency or eczema [5]. Consequently, alternative vaccination strategies including administration of attenuated strains of VACV, recombinant proteins and DNA are being evaluated [6].

Orthopoxviruses, including VACV and variola virus, have two major infectious forms known as the mature virion (MV) and the enveloped virion (EV) [7]. The precursor MV membrane is formed at the initial stage of morphogenesis within specialized areas of the cytoplasm, whereas the EV membrane is derived from modified Golgi or endosomal membranes and encloses the MV [8]. The EV membrane has a role in intracellular trafficking and extracellular spread, whereas the MV membrane fuses with the cell membrane to allow entry of the core into the cytoplasm [9,10]. The viral protein compositions of the two membranes are entirely different and the most effective protein and DNA vaccines induce antibodies to components of both [11-14]. Several MV membrane proteins are known targets of neutralizing antibody: A27 [15,16], A28 [17], D8 [18], H3 [19,20] and L1 [21]. Of these proteins, A27 [22-24], H3 [19] and D8 [25] are involved in virus attachment and A28 [26] and L1 [27] in membrane fusion and virus entry. The MV proteins do not traffic through the secretory pathway of the cell, creating obstacles to their isolation for protein vaccines and presentation for DNA vaccines.

The L1 protein lacks a signal peptide but is myristoylated at the N-terminus and has a C-terminal transmembrane domain [28]. The ectodomain of L1 faces the cytoplasm in intracellular virions and contains three intramolecular disulfide bonds that are formed by VACV encoded redox system [29]. A soluble, recombinant form of L1 was made by attaching a signal peptide to the N-terminus and removing the C-terminal transmembrane domain [13,30]. When expressed in insect cells, the secreted protein was correctly folded and capable of inducing neutralizing antibody. Having shown that L1 could be engineered to traffic through the secretory pathway, we investigated a related approach to improve DNA vaccination. Modifications of the gene encoding L1 included codon optimization for mammalian expression, mutation of glycosylation sites since the viral protein is not glycosylated, addition of a signal peptide for traffic through the endoplasmic reticulum and Golgi apparatus to the plasma membrane, and the further truncation of the C-terminus to remove the transmembrane domain and allow secretion. As shown here, these modifications achieved the goal of increasing surface presentation and secretion and increased the production of neutralizing antibody in mice. Mice inoculated with plasmids expressing any of the recombinant L1 proteins partially protected mice against disease. The present work complements and extends recent reports of Golden and coworkers [31,32] on immunization with an L1 gene that contains an added signal peptide.

## Results

### Addition of a heterologous signal peptide sequence to L1 increases cell surface expression

**Figure 1:**
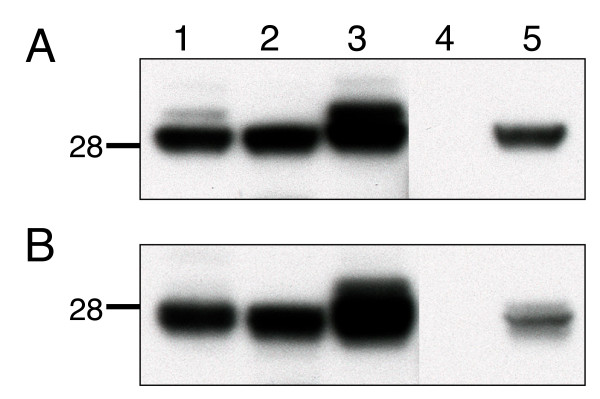
**Expression of modified L1 proteins in BS-C-1 cells determined by Western blotting**. Cells were harvested at 24 h after transfection, lysed, denatured with **(A) **or without **(B) **reducing agent, and subjected to SDS-PAGE. The proteins were transferred to a membrane and probed with polyclonal antibody to L1 and detected by chemiluminescence. Lanes: 1, pL1; 2, pL1op; 3, psL1op; 4, empty vector; 5, VACV-infected lysate. The position of a 28-kDa marker protein is shown on the left.

To initiate this study, we obtained a chemically synthesized L1 gene with N-glycosylation sites removed and codon optimized for expression in mammalian cells. This synthetic L1 gene (L1op) was then further modified by N-terminal addition of DNA encoding the murine Ig κ-chain signal peptide sequence. The original L1 gene, L1op, and the signal peptide modified L1 gene (sL1op) were individually inserted into the eukaryotic expression vector VRC 8400 [33]. Each of the constructs expressed the L1 protein when transfected into BS-C-1 cells as shown by SDS-polyacrylamide gel electrophoresis (PAGE) and Western blotting with a polyclonal L1 antibody (Figure 1A). The major L1R and L1op products migrated to the same position as authentic L1 produced by VACV infection. The sL1op protein was most abundant and appeared as two closely spaced bands, representing full length and signal peptide cleaved versions. In Figure 1B, unreduced proteins were analyzed. Although the pattern remained the same as in Figure 1A, the polypeptides migrated slightly faster relative to the marker proteins, consistent with the presence of intramolecular disulfide bonds [29].

**Figure 2:**
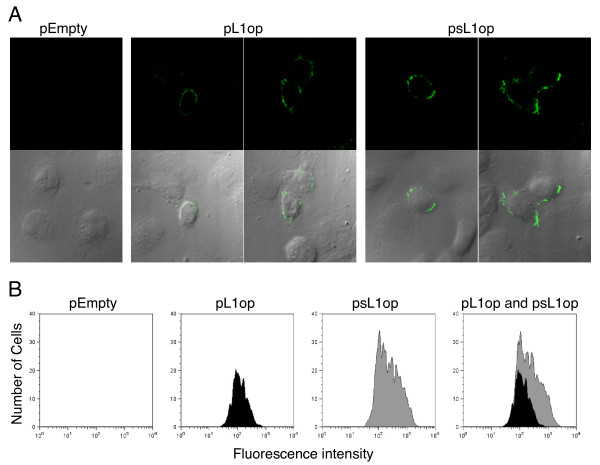
**Cell surface expression of modified L1 proteins determined by confocal microscopy and flow cytometry**. **(A) **BS-C-1 cells were transfected with empty vector, pL1op, or psL1op and stained with anti-L1 mAb (7D11) followed by anti-mouse IgG FITC and viewed by confocal microscopy. Upper panel shows confocal fluorescent images and the lower panel shows a merge of confocal fluorescent and differential interference contrast images. **(B) **BS-C-1 cells were transfected as in panel A. After 24 h, non-permeabilized cells were incubated with MAb 7D11 followed by anti-mouse IgG antibody conjugated to fluorescein isothiocyanate, fixed with paraformaldehyde and analyzed by flow cytometry with gating on L1 positive cells.

Cell surface expression of L1op and sL1op were analyzed by confocal microscopy and flow cytometry of unpermeabilized cells using MAb 7D11, which recognizes correctly folded and disulfide bonded L1 [21,34]. Confocal microscopic analysis indicated that cells expressing sL1op were more frequent and stained more brightly than those expressing L1op (Figure 2A). This impression was supported by more quantitative flow cytometry experiments (Figure 2B). In three separate experiments the mean fluorescence intensity of cell surface L1 expression by sL1op was 2.3, 2.9 and 3.4 times higher than by L1op.

### Increased binding and neutralizing antibodies generated by addition of signal sequence

**Figure 3:**
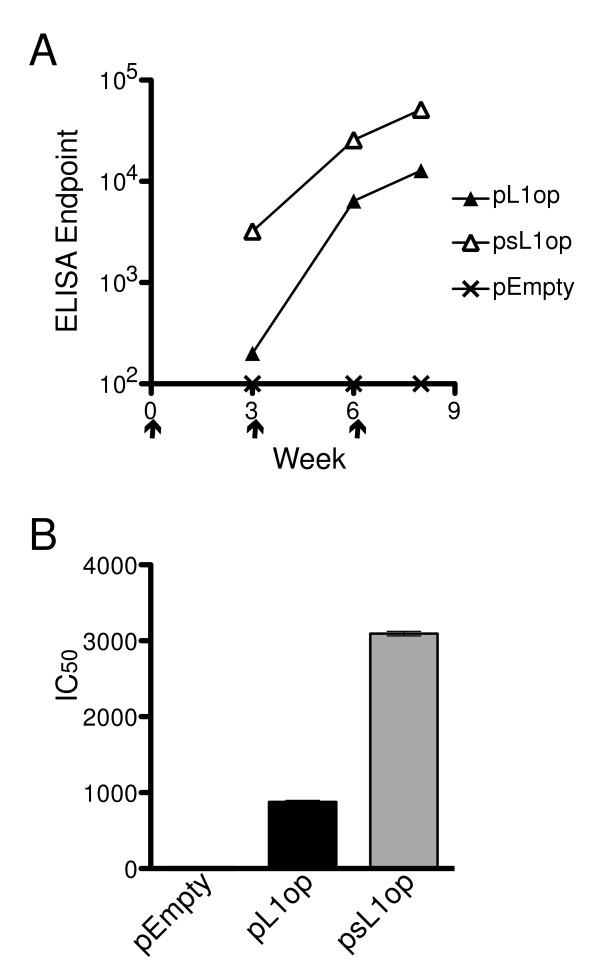
**L1 binding and neutralizing antibodies in sera of mice immunized with pL1op and psL1op**. **(A) **Mice (n = 5) received empty vector, pL1op or psL1op by gene gun on day 0 and after 3 and 6 weeks. Mice were bled at 3, 6 and 8 weeks after the first immunization. Antibody binding to L1 was determined by ELISA. Arrows point to days of immunization. **(B) **Neutralizing antibodies (IC_50_) were measured at 8 weeks using a flow cytometry assay.

Mice were inoculated with plasmids expressing L1op and sL1op to determine whether the different levels of expression translated into higher antibody responses. Three DNA immunizations were administered by gene gun at three-week intervals. L1 binding antibodies were detected in the sera at 3 weeks after the first immunization with L1op or sL1op, however the latter had a 16-fold higher titer (Figure 3A). In both groups of immunized mice, the titers rose after each successive immunization but the difference narrowed so that it was about 4-fold after the second and third immunizations (Figure 3A).

Neutralizing antibody was determined with a well-documented flow cytometry assay using a recombinant VACV that expresses green fluorescent protein [35]. After the third immunization, the neutralizing antibody titer was more than three times higher in those mice that received plasmids expressing sL1op than L1op (Figure 3B). Thus, there was a correlation between increased surface expression and neutralizing antibody titer.

### Expression and immunogenicity of C-terminal truncated forms of L1op and sL1op

**Figure 4:**
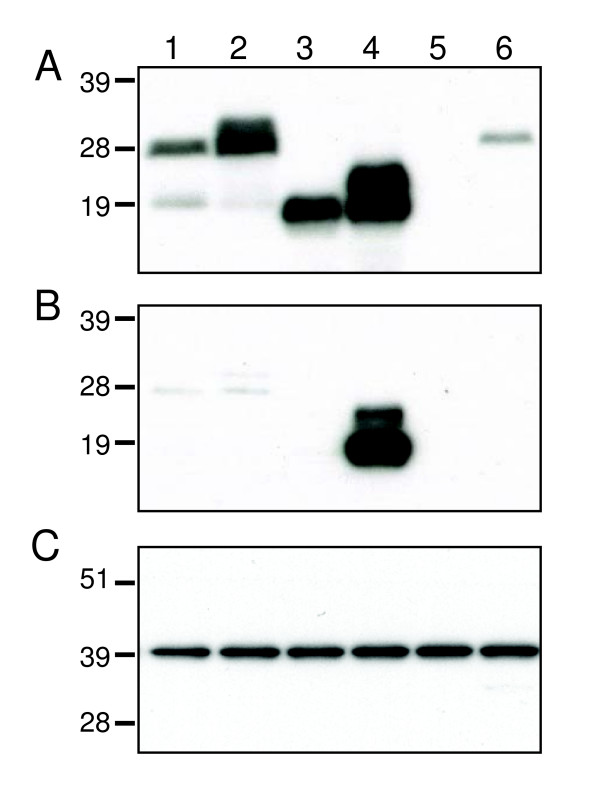
**Expression of truncated L1 proteins detected by Western blotting**. BS-C-1 cells were transfected with plasmids and cells **(A) **and media **(B) **were harvested separately and analyzed by SDS-PAGE followed by Western blotting with polyclonal L1 antibody. In panel C, the Western blot of the cell lysate was probed with antibody to glyceraldehyde 3-phosphate dehydrogenase as a loading control. Proteins were detected by chemiluminescence. Lanes: 1, pL1op; 2, psL1op; 3, pL1optr; 4, psL1optr; 5, empty vector; 6, lysate from VACV-infected cells. The positions and masses in kDa of marker proteins are shown on the left.

Additional constructs were made by truncating the genes encoding L1op and sL1op at amino acid 185 in order to remove the C-terminal transmembrane domain. Analysis of cell extracts by SDS-PAGE and Western blotting indicated that the truncated L1op (L1optr) migrated as a single band, more rapidly that the authentic L1 made by VACV (Figure 4A). The truncated form with a signal peptide (sL1optr) migrated as two bands corresponding to uncleaved and cleaved signal peptide forms (Figure 4A). The cleaved form of sL1optr was also present in the medium with only a small amount of the uncleaved form, whereas neither the truncated form without a signal peptide nor the full-length form with a signal peptide were secreted into the medium (Figure 4B). In Fig. 4C, the Western blot of the cell lysate was probed with antibody to glyceraldehyde 3-phosphate dehydrogenase as a loading control.

**Figure 5:**
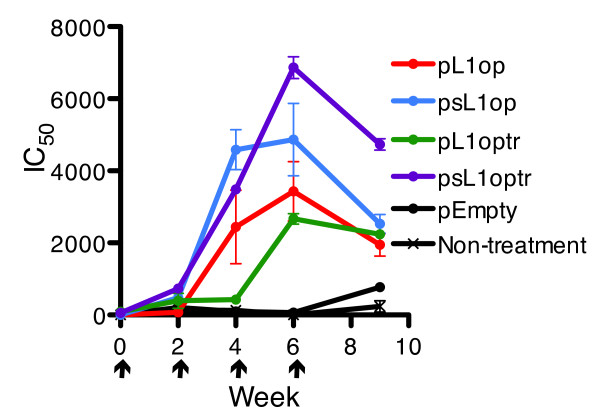
**Effects of signal peptide and presence or absence of transmembrane domain on VACV neutralizing antibodies**. Mice (n = 5) were immunized at 0 time and weeks 2, 4 and 6 with plasmids using a gene gun. Mice were bled at 2 weeks after each of the first three immunizations and 3 weeks after the last. Neutralizing activity was determined by flow cytometry. Arrows point to days of immunization

Mice were inoculated four times by gene gun with plasmids expressing L1optr and sL1optr as well as plasmids expressing full-length versions of L1 in order to compare their immunogenicities. Sera were collected at two weeks after each of the first three immunizations and three weeks after the fourth. Very low neutralizing titers were detected after the first immunization which were boosted after the second and third (Figure 5). The highest neutralizing titers were measured after the third immunization with psL1optr and psL1op, the plasmids containing L1 with signal peptide sequences. The drop in titers after the fourth immunization could be due to the absence of boosting and an additional week before assay.

### Protection of mice by gene gun immunization with plasmids expressing recombinant L1

**Figure 6:**
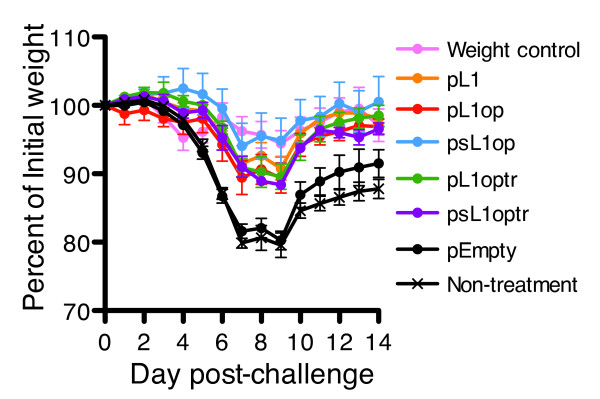
**Partial protection of mice immunized with plasmids expressing L1**. Mice were immunized as described in the legend to Figure 5. At 3 weeks after the last immunization, mice were challenged with 10^4 ^pfu of VACV strain WR intranasally and weighed daily.

Mice immunized four times with each of the 5 constructs were challenged by intranasal immunization [36] with 10^4 ^plaque-forming units of the pathogenic VACV strain WR. Weight loss was used as an indicator of disease [37,38]. Mice immunized with each of the forms of L1 provided statistically significant protection on days 7, 8 and 9 (p < 0.05) compared to the empty vector or no treatment (Figure 6). However, differences between the various forms of L1 were not statistically significant.

## Discussion

The proteins comprising the outer membranes of most viruses traffic through the secretory pathway of the cell where their extracellular domains are glycosylated and disulfide bonds form. Poxviruses are exceptional in that the MV membrane is formed within the cytoplasm and as a consequence the proteins are not normally glycosylated and a virus-encoded redox sytem is required to form disulfide bonds [29]. As part of our laboratory's effort to produce a candidate protein subunit smallpox vaccine, we demonstrated that proper folding and disulfide bond formation of the L1 protein occurs in insect cells if a cleavable signal peptide is appended to the N-terminus and the C-terminal transmembrane domain is removed [13,30]. That study demonstrated that the chaperones and redox enzymes in the endoplasmic reticulum of insect cells could substitute for the cytoplasmic viral enzymes. The purpose of the present study was to determine whether a similar strategy would enhance the presentation of L1 expressed by a DNA vaccine in mammalian cells and enhance immunogenicity in mice. At the time this project was initiated, gene gun immunization with the natural L1 gene had been shown to provide partial protection against challenge with VACV and more complete protection when combined with genes encoding other MV and EV membrane proteins [11,12,39].

We started with a synthetic L1 gene for two reasons. First, our studies (unpublished) and others [40] have shown that codon optimization for mammalian cells can sometimes enhance expression of poxvirus genes. Second, the recombinant L1 protein expressed in insect cells was unnaturally glycosylated [30]. Consequently, the L1 gene was codon optimized and had the three glycosylation sites mutated. Other modifications included addition of an N-terminal signal peptide sequence with or without removal of the C-terminal transmembrane domain. In addition, we used a cytomegalovirus promoter that had been modified for expression in mice [33], since the goal was to test immunogenicity. The transfected synthetic L1op genes were expressed to even higher levels than in VACV-infected cells and the signal peptide sequence produced increased protein on the cell surface. Removal of the transmembrane domain allowed secretion.

Gene gun immunization was used to deliver the recombinant L1 genes as described by Hooper [12]. The highest binding and neutralizing antibody responses were achieved with the proteins containing a signal peptide with or without the transmembrane domain. The differences were more substantial when measured at two weeks after the second and third immunizations compared to three weeks after the fourth immunization, when protection studies were carried out. The protection against weight loss induced by any of the recombinant L1 constructs was statistically significant when compared to empty vector plasmid or untreated mice. However, differences between the individual L1 constructs were not significant. Although antibody responses are important in protection against VACV infection, it is possible that CD8+ T-cells also contributed.

It is useful to compare our study with recent reports of Golden and coworkers [31,32]. This group took a similar approach in attaching a signal peptide to the N-terminus of L1, which retained the transmembrane domain, and also found that this increased induction of neutralizing antibody following gene gun immunization. When challenged with 3 times the LD_50 _of VACV strain IHD-J, all mice died regardless of whether they were vaccinated with modified or unmodified L1. However, the signal peptide modified L1 appeared superior to unmodified L1 when combined with the genes encoding additional MV and EV membrane proteins. Such combinations have been shown by several groups to be important for protection against lethal orthopoxvirus infections of mice and monkeys [11-14,39,41-43].

## Conclusion

Modifications of the VACV L1 gene, including codon optimization, attachment of a signal peptide sequence, and removal of the transmembrane domain can enhance expression and immunogenicity for DNA vaccination.

## Methods

### Cell cultures and viruses

BS-C-1 cells (ATCC CCL-26) were grown in modified Eagle's minimal essential medium (Quality Biologicals, Inc, Gaithersburg, MD) that was supplemented with 10% heat inactivated fetal bovine serum (Hyclone, Logan, UT), 2 mM L-glutamine (Invitrogen, Carlsbad, CA), 100 U/ml of penicillin and 100 μg/ml streptomycin sulfate (Invitrogen). HeLa S3 (ATCC CCL-2.2) suspension cultures were grown in spinner cell Eagle's Minimal Essential Medium (Quality Biologicals, Inc) with the addition of 5% heat-inactivated equine serum (Hyclone). Propagation and purification of VACV strain WR (ATTC VR-1354) has been described [44,45].

### Plasmids and transfection

The natural L1 gene sequence from VACV strain WR was modified by removal of three potential glycosylation sites, mammalian codon optimized and inserted into PCR-Script by GENEART (Regenburg, Germany). A set of modified L1 sequences encoding the murine Ig κ-chain leader sequence [46] and/or truncated after codon 185 were assembled by PCR with terminal PstI and NotI sites and inserted into the corresponding sites of pVRC8400 [33]. For in vitro expression of L1, BS-C-1 cells were transfected with 1.5 μg of plasmid in 10 μl of Lipofectamine™ 2000 (Invitrogen) per well of a 6-well plate.

### Western blot

Twenty-four hours after transfection, cells were washed with phosphate buffered saline and suspended with NuPAGE^® ^LDS Sample Buffer (Invitrogen) and sonicated. The lysates were heated at 70°C for 10 min with or without NuPAGE^® ^Sample Reducing Agent (Invitrogen) and the proteins were resolved by SDS-PAGE in NuPAGE^® ^Bis-Tris gels (Invitrogen). Following transfer to a polyvinylidene difluoride membrane using iBlot PVDF Transfer Stack (Invitrogen), the membrane was incubated with rabbit polyclonal anti-L1 antibody (R180, provided by G. Cohen and R. Eisenberg, University of Pennsylvania) followed by anti-rabbit IgG conjugated to horseradish peroxidase. Bands were visualized with a chemiluminescence detection kit (Pierce, Rockford, IL).

### Confocal microscopy

Unfixed cells were incubated with anti L1 MAb 7D11 [21] provided by Alan Schmaljohn followed by anti-mouse IgG conjugated to fluorescein isothiocyanate and analyzed by confocal microscopy as described [47].

### Flow cytometry

Twenty-four hours after plasmid transfection, cells were washed with phosphate buffered saline and cell suspension made with versene EDTA chelating agent (Invitrogen). The non-permeabilized cells were incubated with MAb 7D11 followed by anti-mouse IgG antibody conjugated to fluorescein isothiocyanate, fixed with 2% paraformaldehyde and analyzed with a FACSCalibur flow cytometer using CellQuest (BD Biosciences) and FlowJo Software (Tree Star, Inc, Ashland, OR).

### Gene gun immunization of mice

Seven weeks old female BALB/c mice were transfected with plasmids by Helios gene gun delivery (BIO-RAD, Hercules, CA). Individual cartridges were prepared with approximately 1 μg of plasmid and 0.5 mg gold particles. Briefly, plasmid DNA, spermidine, CaCl_2 _and 2 micron gold particles (DeGussa, Parsippany, NY) were mixed and washed with ethanol. The mixture were suspended in ethanol and dried onto Tefzel tubing (BIO-RAD). DNA-coated gold particles were delivered with a Helios Gene Gun at 400 pounds per square inch to three non-overlapping sites on the shaved abdomen.

### Antibody binding assay

Antibody binding to purified L1 and VACV particles was carried out by ELISA [17] with some modifications. The 96-well plates (Immulon HB plate, Thermolab System, Hertfordshire, UK) were coated with 100 μl/well of affinity-purified L1 protein (600 ng/ml of phosphate buffered saline) and incubated ~24 h at 4°C. Following incubation with diluted sera followed by anti-mouse IgG-peroxidase (Roche, Branchburg, NJ), the plates were reacted with BM Blue substrate (Roche). The plates were read at wavelengths 370 nm and 492 nm using SpectraMax M5 Microplate Reader and SoftMaxPro Software System (Molecular Devices, Sunnyvale, CA). The endpoint was 0.1 absorbance unit after subtraction of the background absorbance of serum incubated in wells without protein.

### Neutralization assay

Purified VACV expressing enhanced green fluorescent protein [35] was incubated with diluted serum in a 96-well plate for 1 h at 2.5 × 10^4 ^plaque forming units/well. HeLa S3 cells were treated with cytosine arabinoside for 10–15 min and then 1 × 10^5 ^cells were added to each well and the plates incubated for 16–18 h in a 37°C CO_2 _incubator. Incubated cells were fixed with 2% paraformaldehyde in phosphate buffered saline and analyzed on a FACSCalibur flow cytometer using CellQuest and FlowJo Software. IC_50 _values were calculated using PRISM software (GraphPad, La Jolla, CA)

### Statistical methods

p-value was determined by t-test using PRISM software (GraphPad).

## Competing interests

The authors declare that they have no competing interests.

## Authors' contributions

KS participated in the design of the study and was the primary person involved in acquisition, analysis and presentation of the data. KRI assisted in the gene gun vaccination. LSW contributed to the design of the study and analysis of the data. BM participated in the design of the study, evaluation of the data and writing the manuscript.
